# The social determinants of health associated with cardiometabolic diseases among Asian American subgroups: a systematic review

**DOI:** 10.1186/s12913-022-07646-7

**Published:** 2022-02-25

**Authors:** Lucy Y. Min, Rehnuma B. Islam, Nikhila Gandrakota, Megha K. Shah

**Affiliations:** 1 University of Miami Miller School of Medicine, Miami, FL USA; 2 Department of Family and Preventive Medicine, Emory University School of Medicine, Atlanta, GA USA

**Keywords:** Cardiometabolic disease, Diabetes, Hypertension, Asian American, Social determinants of health

## Abstract

**Background:**

Asian Americans represent one of the fastest-growing immigrant groups in the U.S. and are at high risk for cardiometabolic diseases (CMDs), including type 2 diabetes, hypertension, coronary artery disease, and stroke. Despite the growth of Asians in the U. S, there is a gap in understanding the heterogeneity of CMDs across Asian subgroups and how these might be affected by the social determinants of health (SDOH), or the environment in which people live and work.

**Objective:**

The purpose of this systematic review is to examine the current literature on CMDs among Asian Americans and identify the SDOH that are associated with the incidence and/or prevalence of CMDs among specific Asian subgroups.

**Methods:**

PubMed, Embase, Web of Science were searched for articles published in Jan 2000-Nov 2020. The reproducible strategy yielded 2732 articles. The articles were reviewed based on the following inclusion criteria: (1) observational study published in the U.S., (2) adult population includes specific Asian subgroups, (3) exposures include SDOH, and (4) outcomes include a CMD, defined as type 2 diabetes, hypertension, coronary artery disease, or stroke.

**Results:**

In this review, 14 studies were identified and organized into four key themes: acculturation (*n* = 9), socioeconomic status (SES) (*n* = 6), social context (*n* = 2), and health literacy (*n* = 1). The most represented Asian subgroups in the literature were Chinese, Filipino, and South Asians. Acculturation was the most described social factor in the included reviews. Seven studies found associations between higher acculturation levels and higher prevalence of CMD. However, the measure of acculturation varied by study and included various combinations of the country of birth, number of years residing in the U.S., and English proficiency. The effects of SES, measured as income level and educational attainment, varied by racial subgroups. One study found that higher levels of education were associated with CMD among South Asians.

**Conclusion:**

Acculturation, SES, social context, and health literacy impact the risk of CMD among Asian Americans; these vary across subgroups. Future research disentangling SDOHs on the risk of CMDs by Asian subgroup is necessary to provide better informed preventive practices and interventions.

**Supplementary Information:**

The online version contains supplementary material available at 10.1186/s12913-022-07646-7.

## Introduction

The United States has 18.9 million (5.9% of the U.S. population) Asian Americans, defined as a person who belongs to the Asian race as “having origins in any of the original peoples of the Far East, Southeast Asia, or the Indian subcontinent” including, for example, Cambodia, China, India, Japan, Korea, Malaysia, Pakistan, the Philippine Islands, Thailand, and Vietnam, between others [1, 2]. The Asian population is the second-fastest-growing group in the U.S. and the largest immigrant group, projected to increase by 128%, reaching 46 million by 2060 [3–5]. In 2019, Chinese Americans comprised the largest percentage (23%) of Asians in the U.S., followed by Asian Indians (20%) and Filipinos (18%) [4]. Vietnamese, Koreans, and Japanese comprised more than 1 million each among the U.S. Asians [4].

Evidence shows that Asians are at a higher risk of cardiometabolic disease (CMD) at a lower BMI than other races [6–8]. Multiple studies also showed that Asian immigrants have an increased risk of cardiometabolic diseases, which includes type 2 diabetes, hypertension, coronary artery disease, stroke [9–11]. According to WHO, the social determinants of health (SDOH) are “the circumstances in which people are born, grow, live, work, and age, and the systems put in place to deal with illness” [12]. A recent scientific report from AHA mentions education, income, and occupation to be the most common social determinants influencing cardiometabolic health [13]. It also reiterates the importance of addressing the specific health needs of the rapidly increasing Asians as they are also disproportionately burdened with poor health across a variety of outcomes [13]. However, despite the vast diversity among the Asian subgroups by origin, culture, genetics, immigration patterns, socioeconomic factors, lifestyle, and cardiometabolic risk, they are still studied for medical and research purposes under a single group “Asian”.

Previous research showed the disparities in education and income level of Asian American subgroups with Asian Indians at higher and Koreans and Vietnamese at lower levels of these social determinants of health [14]. The pattern of immigration has also been different among the various Asian subgroups with the majority of the Asian Indians migrating to the U.S. after the 1965 Immigration Act, while the Chinese initially moved between 1820 and 1880 [14]. Immigration patterns also varied among Koreans, Vietnamese, Japanese. and Filipinos too [14]. There have also been differences in the regions these subgroups settled in the U.S., their income, and education levels [14]. Hence, grouping all these Asian subgroups together as “Asian” masks the heterogeneity in the social factors which influence their cardiometabolic health.

The purpose of this systematic review is to critically review the existing research that focuses on the relationship between SDOH and CMDs among various Asian subgroups and describe the most common SDOHs that are associated with CMD among the disentangled Asian subgroups. We hypothesize that the SDOHs vary and do not uniformly affect subgroups. This review will identify future areas for targeted research and interventions to address the SDOH among specific Asian groups.

## Methods

This systematic review was conducted according to the guidelines of the Preferred Reporting Items for Systematic Reviews and Meta-Analyses (PRISMA) [15]. This review protocol was registered in PROSPERO (CRD42021221791).

### Search strategy

We conducted electronic searches in the following databases: PubMed (MEDLINE), Embase, Web of Science in November 2020. The search was limited to articles published between January 2000 and November 2020. The full search strategy with keywords is listed in Additional File 1.

### Study selection

To determine the eligibility of inclusion, the studies were initially screened based on title and abstract by two independent reviewers. Studies deemed eligible for full-text review were considered for inclusion by two independent reviewers. The title, abstract, and full-text screening were conducted on Covidence, an online systematic review software [16]. Disputes in any stage of the review were decided by the senior author.

### Eligibility criteria

For a study to be included in the review, it had to meet the following inclusion criteria: (1) observational study conducted in the United States and published between January 2000 and November 2020; (2) adult population includes at least one specific Asian American subgroup and reported data is stratified by racial/ethnic subgroups; (3) examines at least one social determinant of health and its association to a cardiometabolic disease, which includes type 2 diabetes, hypertension, coronary artery disease, stroke; (4) outcomes include a lab-measured or self-reported physician diagnosis of a CMD, defined as type 2 diabetes, hypertension, coronary artery disease, stroke. Studies were excluded if it was conducted outside of the U.S., included an intervention, included pediatric patients, did not stratify by specific Asian subgroups, and/or did not examine a SDOH.

### Data extraction

Three reviewers independently extracted information from the included studies using the PECO format: population, exposure, comparison, outcome [17]. The data extraction included: first author name, year of publication, description of study setting, study design, the Asian subgroup that was included in the study population, the social factor(s) examined as an exposure, the cardiometabolic disease(s) included as an outcome, and the findings of the study. The final data extraction was reviewed and synthesized by the first author. Due to the observational design of the included studies, the extracted data was described and compared qualitatively in this review.

### SDOH classifications of studies

**Fig. 1:**
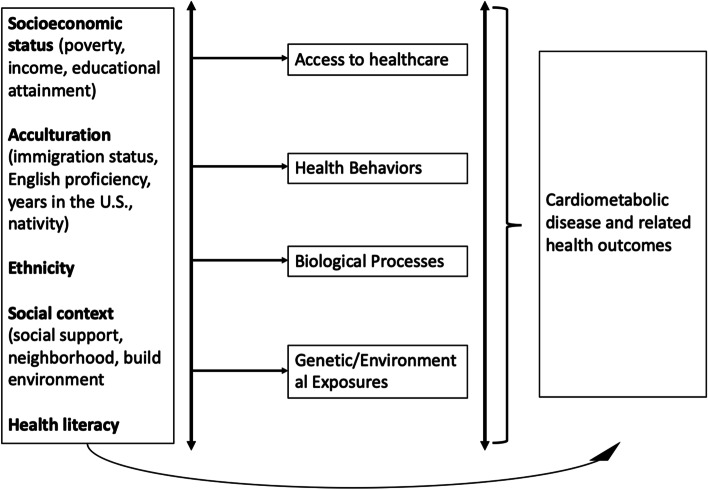
Framework for Social Determinants of Health adapted from Healthy People 2030 SDOH and WHO

We adapted the Healthy People 2030 SDOH framework and the WHO framework on social determinants of health to guide our review, definitions, and rationale for inclusion for each of the SDOH exposures (see Fig. 1) [18, 19]. Based on this adapted framework and discussion among the co-authors, the studies were classified into examining one of the following four social factors: acculturation, socioeconomic status (SES), social context, and health literacy. Acculturation is roughly defined as the process by which individuals adopt the customs, behaviors, and beliefs of the host culture, in this case, the U.S. The measure of acculturation varied between studies. We broadly defined this as country of birth (i.e. nativity), years of residence in the U.S., and English proficiency as measures of acculturation for inclusion. SES is broadly defined as a combination of education, income, and occupation. In this review, we separated income level and educational attainment as two separate subcategories for a closer examination of these social factors. Social context is defined as any aspects of social support, cohesion, or neighborhood factors included in studies. Health literacy is defined as the “degree to which individuals have the ability to find, understand, and use information and services to inform health-related decisions and actions for themselves and others [20].

### Quality assessment

All included studies were assessed by two independent reviewers using the National Heart, Lung, and Blood Institute (NHLBI) Study Quality Assessment Tool for Observational Cohort and Cross-sectional Studies [21]. The studies were evaluated using the 14-item list assessing the research question, study population, sample size, timeframe, exposure measures and assessment, outcome measures and assessment, follow-up rate, and statistical analysis [21].

## Results

**Fig. 2:**
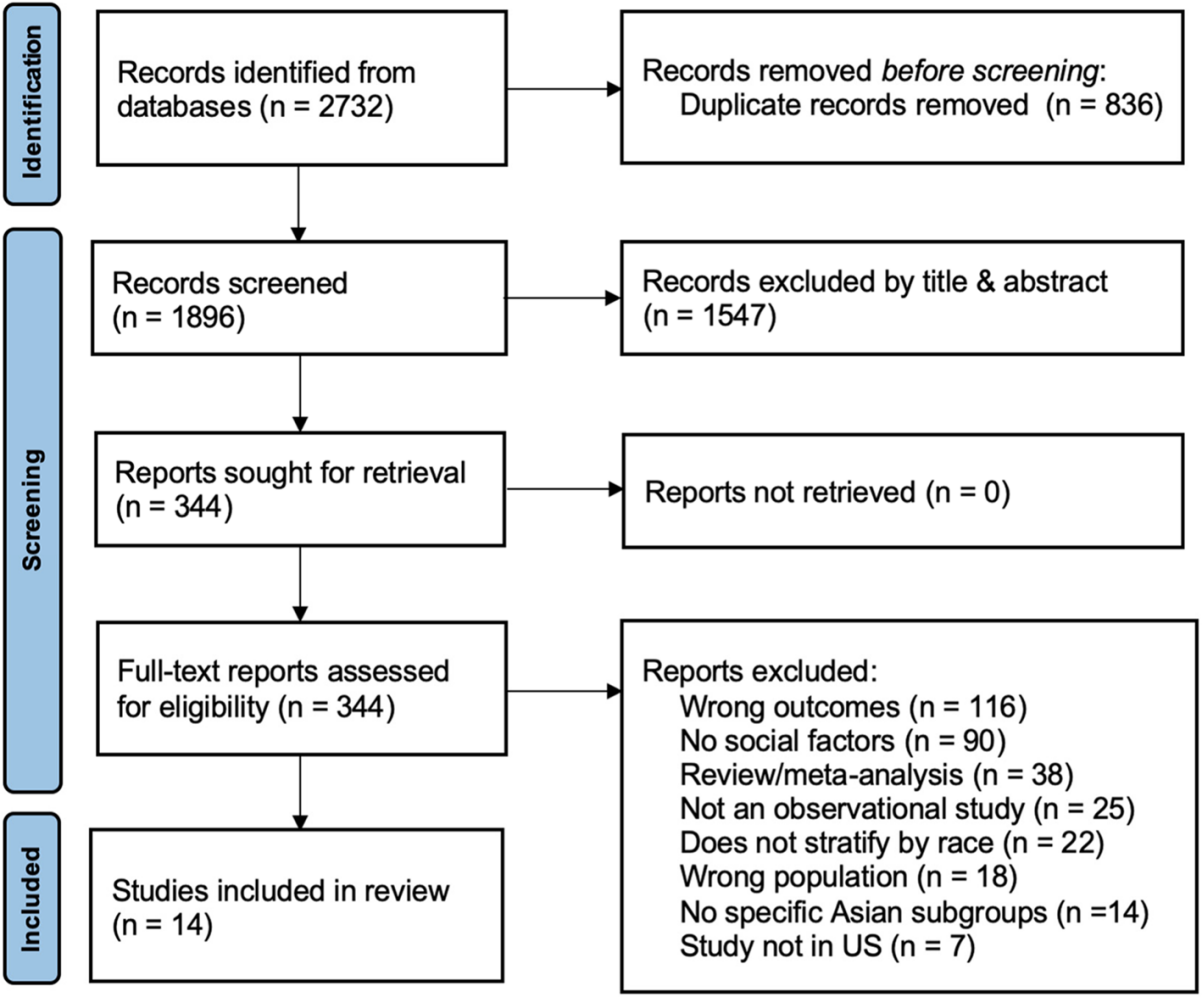
PRISMA flow diagram

The initial result of our combined searches from PubMed, Web of Science, and Embase yielded 2732 articles of which 836 were excluded as duplicates. This left 1896 studies that underwent title and abstract screening of which 1547 were excluded. The full-text review was completed for 344 studies. Of these articles, 329 were excluded due to wrong outcomes, not including social factors as an exposure variable, the study being a review or meta-analysis, not stratifying data by race, the wrong population being studied, including no specific Asian subgroup, or study not being completed in the United States. Fourteen studies were considered eligible for inclusion in the systematic review. See Fig. 2 for the PRISMA diagram highlighting the study selection process.

### Study characteristics

Studies deemed eligible for inclusion examined SDOH associated with CMDs among Asian American subgroups. Descriptive information about the studies is provided in Table 1. The studies included in this systematic review consisted of all observational, cross-sectional studies (*n* = 14) (Table 1). Of the 14 included studies, the main CMD outcomes were diabetes (*n* = 7), hypertension (*n* = 6), and both diabetes and hypertension (*n* = 1) (Table 1). The represented Asian subgroups in the selected literature were Chinese (*n* = 6), Filipino (*n* = 6), South Asian (*n* = 5), Korean (*n* = 4), Vietnamese (*n* = 2), and Japanese (*n* = 2) (Table 2). Four main themes of SDOH identified through this review were acculturation (*n* = 9), SES (*n* = 6), social context (*n* = 2), and health literacy (*n* = 1) (Table 3). While the outcomes and exposures varied by study, all the studies included an assessment and adjustment for potential confounders and met these criteria of the NHLBI quality assessment for observational studies (quality assessment included in Table 1).

**Table 1:** Table of included studies and summary of findings

Author (Year)	Setting	Study Design	Included Asian subgroup	Social factor examined	CMD	Impact on outcome	Quality assessment rating
Lee et al. (2020) [22]	NHIS	Cross-sectional study	Chinese, Filipino, Asian Indian, Other Asians (Korean, Japanese, Vietnamese, and other Asian subgroups)	Acculturation	Diabetes, Cardiovascular disease (coronary heart disease, stroke)	Compared to U.S.-born white adults, the prevalence of type 2 diabetes was significantly greater among foreign-born Asians living in the U.S. for at least 15 years (OR = 1.3 [95% CI: 1.2–1.5]). Among Asians, foreign-born Asian Indians living in the U.S. for ≥15 years had highest odds of type 2 diabetes compared to U.S.-born whites followed by foreign-born Filipinos with ≥15 years of residency.	Fair
Bayog et al. (2018) [23]	CHIS from 2011 to 2012	Cross-sectional study	Filipino	Acculturation	Diabetes, hypertension	Nativity was significantly associated with hypertension and diabetes. Long-term immigrant Filipinos were 2.8 times (p < .0005) more likely to have hypertension and 4 times (*p* < .0005) more likely to have diabetes compared to second-generation Filipinos. Recent immigrants were less likely to have CMD compared to long-term immigrants but more likely than second-generation	Fair
Ursua et al. (2013) [24]	Baseline data from the Project AsPIRE in the NYC and NJ area	Cross-sectional study	Filipino	Acculturation	Hypertension	Longer residence in U.S. was associated with hypertension status among Filipino immigrants. No association between language spoken and hypertension.	Fair/good
Ma et al. (2017) [25]	Recruited from 8 Filipino community-based organizations in the PA and NJ region	Cross-sectional study	Filipino	Acculturation, Education	Hypertension	Filipinos who resided in the U.S. for 20–30 years were 3.73 times more likely to have hypertension than Filipinos who lived in the U.S. for less than 19 years. Filipinos with hypertension typically were more likely to have a college degree than Filipinos without hypertension.	Fair
Yi et al. (2016) [26]	NYC Community Health Survey	Cross-sectional study	Chinese, South Asian	Acculturation, SES (Income, Education)	Hypertension	No statistical significance in association between years lived in U.S. and hypertension. Chinese and South Asian immigrants with hypertension were significantly less likely to speak English at home. Compared to Whites counterparts, foreign-born Chinese adults with hypertension were of a much lower SES profile. South Asians with hypertension were more likely to have a college education than White counterparts, while Chinese with hypertension were less likely to have a college education.	Fair
Kim et al. (2000) [27]	Two types of community-based sites in MA: Korean churches and Korean grocery stores	Cross-sectional study	Korean	Acculturation, SES (Education)	Hypertension	Korean Americans with low English proficiency were more likely to be hypertensive. Those with education less than high school were more likely to have hypertensive than those with education greater than high school.	Good
Huang et al. (2015) [28]	CHIS from 2007 to 2009	Cross-sectional study	Chinese, Filipino, South Asian, Vietnamese, Korean, Japanese	Acculturation	Diabetes	The association of acculturation and diabetes varied based on ethnicity and gender. Among Filipinos, women who only spoke English at home were less likely to have diabetes, while men who only spoke English at home were more likely to have diabetes. Chinese men who spoke English at home were more likely to have diabetes.	Fair
Kandula et al. (2008) [29]	MESA recruited from Baltimore, MD; Chicago, IL; Forsyth Co, NC; Los Angeles, CA; NY, NY; St Paul, MN	Cross-sectional study	Chinese	Acculturation	Diabetes	Among Chinese participants, there was no significant association between acculturation score and diabetes prevalence.	Good
Yang et al. (2007) [30]	Mail survey in Michigan	Cross-sectional study	Korean	Acculturation	Diabetes, heart disease	No statistically significant trend in prevalence of diabetes in relation to length of residence in the U.S. for both men and women.	Fair
Boykin et al. (2011) [31]	MESA recruited from Baltimore, MD; Chicago, IL; Forsyth Co, NC; Los Angeles, CA; NY, NY; St Paul, MN	Cross-sectional study	Chinese	SES (Income, Education)	Hypertension	No statistically significant association with income level and hypertension among Chinese women. No statistically significant association with income level and diabetes among Chinese men. Higher level of education was associated with lower prevalence of diabetes & hypertension for both Chinese women and men, but these trends were not statistically significant.	Fair/good
Shah et al. (2015) [9]	MASALA- Two clinical sites: San Francisco Bay Area through UCSF and greater Chicago area through Northwestern University	Cross-sectional study	South Asian	SES (Income, Education)	Diabetes	Lower income (<$40,000 annually) and having less than a bachelor’s degree were associated with a greater prevalence of diabetes among South Asians although the tests of heterogeneity were not statistically significant (*p* = 0.11 and *p* = 0.26 respectively).	Good
Sentell et al. (2011) [32]	Hawai’i Health Survey conducted by the Hawai’i State Dept of Health, Office of Health Status Monitoring	Cross-sectional study	Filipino, Japanese, other AAPI	SES (Education), Health Literacy	Diabetes	Japanese Americans with education less than high school were less likely to have diabetes compared to those with education more than high school. Similar nonsignificant trends were observed in Filipino Americans. Low health literacy was significantly associated with diabetes for Native Hawaiians and Japanese (p < 0.05). Odds ratio of low health literacy predicting diabetes was 1.80 for Filipino and 1.78 for Japanese.	Fair
Lagisetty et al. (2016) [33]	MASALA- Two clinical sites: San Francisco Bay Area through UCSF and greater Chicago area through Northwestern University	Cross-sectional study	South Asian (India, Pakistan, Bangladesh, Nepal, Sri Lanka)	Social context	Diabetes	In the overall sample, no association between social cohesion and prevalence of hypertension or type 2 diabetes. But in South Asian women, higher perceived neighborhood social cohesion was associated with decreased prevalence of hypertension.	Good
Lu et al. (2019) [34]	Baseline survey data from Asian American Liver Cancer Prevention Program was used for this ancillary study in Washington, DC	Cross-sectional study	Chinese, Korean, Vietnamese	Social context	Hypertension	Chinese participants with high social support were 64% less likely to have hypertension as compared to those who had low social support (OR 0.36, 95% CI 0.15, 0.87). Among Korean and Vietnamese groups, no significant difference in hypertension status was found for various psycho-logical measures.	Good

*NHIS* National Health Interview Survey, *CHIS* California Health Interview Survey, *AsPIRE* Asian American Partnership in Research and Empowerment, *MESA* Multi-Ethnic Study of Atherosclerosis, *MASALA* Mediators of Atherosclerosis in South Asians Living in America

**Table 2:** Represented Asian subgroups in the literature selected for the systematic review

	Chinese	Filipino	South Asian^a^	Korean	Vietnamese	Japanese	Other Asian
Lee et al. (2020) [22]	✓	✓	✓^b^				✓^c^
Bayog et al. (2018) [23]		✓					
Ursua et al. (2013) [24]		✓					
Ma et al. (2017) [25]		✓					
Yi et al. (2016) [26]	✓		✓				
Kim et al. (2000) [27]				✓			
Huang et al. (2015) [28]	✓	✓	✓	✓	✓	✓	
Kandula et al. (2008) [29]	✓						
Yang et al. (2007) [30]				✓			
Boykin et al. (2011) [31]	✓						
Shah et al. (2015) [9]			✓				
Sentell et al. (2011) [32]		✓				✓	✓
Lagisetty et al. (2016) [33]			✓				
Lu et al. (2019) [34]	✓			✓	✓		
**Total**	**6**	**6**	**5**	**4**	**2**	**2**	**2**

^a^ South Asian includes immigrants from India, Pakistan, Bangladesh, Nepal, and Sri Lanka

^b^ Lee et al. included only Asian Indians, but for simplicity of the table, they are listed under the South Asian column

^c^ Lee et al. listed “Other Asians” as Korean, Japanese, Vietnamese, and other Asian subgroups

**Table 3:** Themes of SDOH associated with CMDs among Asian subgroups

	Acculturation	Socioeconomic Status	Social context	Health Literacy
Lee et al. (2020) [22]	✓			
Bayog et al. (2018) [23]	✓			
Ursua et al. (2013) [24]	✓			
Ma et al. (2017) [25]	✓	✓		
Yi et al. (2016) [26]	✓	✓		
Kim et al. (2000) [27]	✓	✓		
Huang et al. (2015) [28]	✓			
Kandula et al. (2008) [29]	✓			
Yang et al. (2007) [30]	✓			
Boykin et al. (2011) [31]		✓		
Shah et al. (2015) [9]		✓		
Sentell et al. (2011) [32]		✓		✓
Lagisetty et al. (2016) [33]			✓	
Lu et al. (2019) [34]			✓	
**Total**	**9**	**6**	**2**	**1**

### Acculturation

Of the 14 studies, seven were found to examine the association of acculturation among diabetes (*n* = 2), hypertension (*n* = 3), cardiovascular disease and diabetes (*n* = 1) and both diabetes and hypertension (*n* = 1) (Table 1). The classification of acculturation varied by study; each study defined acculturation using a combination of the following factors: country of birth, years lived in the U.S., language spoken at home, English proficiency, and generational status, which is defined as whether the population is foreign-born (1st generation) or US-born (2nd generation) (Table 4).

**Table 4:** Acculturation definitions by study

	Country of birth	Years lived in the U.S.	Language spoken at home	English proficiency	Generational Status
Lee et al. (2020) [22]	✓	✓			
Bayog et al. (2018) [23]		✓			
Ursua et al. (2013) [24]		✓	✓		
Ma et al. (2017) [25]	✓	✓		✓	
Yi et al. (2016) [26]		✓	✓		
Kim et al. (2000) [27]		✓		✓	
Huang et al. (2015) [28]	✓		✓		✓
Kandula et al. (2008) [29]			✓		
Yang et al. (2007) [30]		✓			

Four studies found that for Asian immigrants, longer residence in the U.S. was associated with a higher prevalence of CMDs. Lee et al. identified the association between nativity and prevalence of diabetes and cardiovascular disease, which included coronary heart disease and stroke, among Chinese, Filipino, Asian Indians, and other Asians compared to U.S.-born whites [22]. Nativity was used as an indicator for acculturation; nativity was defined as a combination of the country of birth (U.S.-born vs foreign-born) and length of U.S. residency [22]. When adjusted for age, sex, obesity, and other sociodemographic factors, foreign-born Asians were more likely to have diabetes (OR 1.7, 95% CI 1.5–1.9) but less likely to have cardiovascular disease (OR 0.7, 95% CI 0.6–0.8) compared to U.S.-born whites [22]. Among foreign-born Asians, longer residence in the U.S. was associated with a higher prevalence of diabetes and cardiovascular disease; however, this was not a statistically significant trend [22]. Among the Asian subgroups, foreign-born Asian Indians living in the U.S. for 15+ years had the highest prevalence of diabetes compared to U.S.-born whites (OR 2.3, 95% CI 1.0–1.6) [22]. Foreign-born Filipino Americans living in the U.S. for 15+ years had the second-highest prevalence of diabetes compared to U.S.-born whites (OR 2.0, 95% CI 1.7–2.4) [22].

Of the four studies that found the association between longer residence in the U.S. and higher prevalence of CMDs, three of the studies examined this trend in Filipinos. Bayog et al. studied the influence of nativity on chronic health conditions, including hypertension and diabetes, among Filipino Americans [23]. Participants were grouped based on nativity into three categories: recent immigrant (0–14 years in U.S.), long-term immigrant (15+ years in U.S.), or second generation (U.S.-born) [23]. Compared to second generation Filipino, long-term immigrant Filipino were more likely to have hypertension (OR 2.8, 95% CI 1.9–4.2) and diabetes (OR 4.0, 95% CI 2.2–7.4) [23]. Recent immigrant Filipinos were more likely to have hypertension and diabetes than second generation Filipinos but less likely than long-term immigrant Filipinos [23].

Two of these studies found an association between longer residence in the U.S. and CMDs but found no association with English proficiency. Ursua et al. studied Filipino Americans living in New York and New Jersey to determine the prevalence of hypertension and examine the associated risk factors [24]. Two of the risk factors measured were years lived in the U.S. (≤5 years vs. 6–15 years vs. ≥15 years) and language spoken (English vs. no English) [24]. Hypertension status was significantly associated with longer residence in the U.S. Filipino immigrants who lived in the U.S. for 15 or more years were 1.6 times more likely to have hypertension when compared to Filipino immigrants who lived in the U.S. for 5 or less years (*p* < 0.05) [24]. There was no significant association between the language spoken and the prevalence of hypertension [24]. Ma et al. studied Filipino Americans living in Pennsylvania and New Jersey to identify the risk factors associated with hypertension [25]. They assessed acculturation based on country of birth, the number of years lived in the U.S., English proficiency (speaking and reading), language spoken at home, and food preference [25]. There was marginal significance in the increased prevalence of hypertension among Filipinos who have lived in the U.S. for 20–30 years compared to Filipinos who lived in the U.S. for less than 19 years (OR 3.73, 95% CI 0.90–15.40) [25]. There was no statistical significance in the language spoken at home and hypertension status [25].

In contrast, one study found no statistical significance with the length of residence in the U.S. but an association between the language spoken at home and CMDs. Yi et al. examined hypertension risk factors among Chinese and South Asian immigrants living in New York City [26]. Acculturation was measured by language spoken at home (English vs. no English) and years in the US (< 10 years vs. 10+ years) [26]. There was no statistically significant difference with the years lived in the US among hypertensive Chinese, South Asian, and non-Hispanic white participants [26]. They found that both Chinese and South Asian immigrants with hypertension were significantly less likely to speak English at home than their non-Hispanic White counterparts [26]. The percentage of Chinese immigrants with hypertension who spoke English at home was 4.0%, while 33% of South Asian immigrants with hypertension spoke English at home and 83.7% of their non-Hispanic white counterparts spoke English at home [26].

One study found that limited English proficiency was associated with a higher prevalence of CMDs among Koreans living in the U.S. Kim et al. studied the prevalence of hypertension and its associated risk factors among Korean Americans living in Maryland [27]. Acculturation was measured using English proficiency and the language choice of media for seeking information or entertainment (English vs Korean mass media) [27]. Korean Americans who spoke English poorly were more likely to be hypertensive than those whose English-speaking ability was good (OR 1.77, 95% CI 1.12–2.80) [27]. It was also found that the prevalence of hypertension in Korean Americans was much higher compared to the prevalence in Koreans residing in Korea [27].

One study found that the association of acculturation, specifically generational status and language spoken at home, with prevalence of diabetes varied based on ethnicity and gender. Huang et al. examined the association in age-adjusted type 2 diabetes (T2DM) among Asians living in California [28]. Acculturation was defined as generational status and language spoken at home [28]. They found that second-generation Asian men and first-generation Asian women had higher T2DM prevalence compared with their white counterparts; this was a trend also observed among Chinese and Filipino men, and Filipina and Korean women [28]. Moreover, Filipinas who spoke only English at home had lower odds of T2DM than those Filipinas who did not speak English at home (OR 0.3, 95% CI: 0.1–1.0) while the relationship was reversed among Filipino men, with English spoken at home related to a higher odds of T2DM (OR 3.2, 95% CI 1.0–10.1) [28]. Additionally, Chinese men who spoke only English at home faced higher odds of reporting T2DM than those who spoke another language at home (OR 3.7, 95% OR: 1.5–9.0) [28].

Two studies found no statistical significance in the association between acculturation and CMDs among Korean and Chinese Americans. Kandula et al. examined the association between acculturation and diabetes among Hispanic and Chinese participants [29]. Each participant was given an acculturation score based on nativity (U.S.-born vs. Foreign-born), years in the U.S., and language spoken at home [29]. They found no significant association between acculturation score and diabetes prevalence among Chinese American participants [29]. Yang et al. examined acculturation status and prevalence of chronic diseases, including diabetes and heart disease, among Korean Americans living in Michigan [30]. Acculturation was measured as the length of residence in the U.S. [30]. There was no statistically significant trend in the prevalence of both diabetes and diabetes in relation to the length of residence in the U.S. for both men and women [30].

### Socioeconomic status

Three studies examined income level and six studies examined educational attainment as a social factor.

#### Income level

Three studies examined the association of income levels with diabetes (*n* = 1), hypertension (*n* = 1), and both hypertension and diabetes (*n* = 1). The study by Yi et al., which was previously mentioned and described for acculturation, found that compared to non-Hispanic White adults with hypertension, foreign-born Chinese adults with hypertension were of a much lower socioeconomic profile (< 200% of Federal Poverty Line) [26]. Boykin et al.’s study observed inverse socioeconomic gradients in hypertension and diabetes in White and Black women but associations were weaker or absent in Hispanic and Chinese women [31]. Additionally, there was no association of income with diabetes in White men (OR 0.94, 95% CI 0.75–1.17) and Chinese men (OR 1.27, 95% CI 0.94–1.71) [31]. Associations of income with hypertension were not statistically significant in any racial/ethnic group in this study [31]. Lastly, Shah et al.’s study found that lower income (<$40,000 annually) was associated with a greater prevalence of diabetes in South Asians although the tests of heterogeneity were not statistically significant [9].

#### Educational attainment

Six of the selected studies examined the association of education status (and diabetes (*n* = 2), hypertension (*n* = 3), and both hypertension and diabetes (*n* = 1). The cutoff for education status varied by study. Among the 6 papers reviewed, one demonstrated higher levels of education were associated with CMD. However, 4 found no association or a weak association, while one demonstrated an association between lower levels of education and CMD .

Kim et al., which was previously described for its observation of acculturation, found (among 606 adults) that educational attainment was associated with hypertension among Korean Americans. Those with education less than high school were statistically significantly more likely to be hypertensive when compared to those with education more than high school (OR 1.58, 95% CI 1.00–2.50) [27].

The study by Ma et al., which was previously described for its observation of acculturation, also found that education level was significantly different among hypertensive and non-hypertensive groups of Filipino Americans and that participants with hypertension were more likely to have obtained a college degree or higher [25].

Yi et al.’s study also that foreign-born Chinese with hypertension were 3 times less likely to have a college education (14.7 vs. 43.2, *p* < 0.001) compared non-Hispanic White adults [26]. Boykin et al. did not find a statistically significant trend in the association between education and prevalence of diabetes among Chinese men and women [31]. In a logistic regression analysis, for Chinese women, higher levels of education were insignificantly associated with a lower prevalence of diabetes and hypertension (OR diabetes 0.83, 95% CI 0.65–1.08; OR hypertension 0.95, 95% CI 0.79–1.15) [31]. Similar trends were observed among Chinese men; higher levels of education were insignificantly associated with a lower prevalence of diabetes and hypertension (OR diabetes 0.82, 95% CI 0.63–1.08; OR hypertension 0.84, 95% CI 0.68–1.04) [31].

Shah et al.’s study found that having less than a bachelor’s degree was associated with a greater prevalence of diabetes in South Asians although the tests of heterogeneity were not statistically significant (*p* = 0.26) [9]. In contrast, Yi et al. found that South Asian immigrants with hypertension were more likely to have a college education when compared to their non-Hispanic White counterparts [26]. This analysis was however statistically insignificant as well [26].

Lastly, the study by Sentell et al. found statistical significance in that Japanese-Americans with education less than high school were less likely to have diabetes when compared to those with education more than high school (OR 0.30, 95% CI 0.09–0.97) [32]. However, when looking at Japanese participants with only high school compared to more than high school, they were more likely to have diabetes (OR 1.36, 95% CI 0.78–2.38); this however was not statistically significant [32]. This similar nonsignificant trend was observed in Filipino Americans [32].

### Social context

Two of the studies examined the effects of social context on the prevalence of hypertension and type 2 diabetes. Lagisetty et al. investigated the association between perceived neighborhood social cohesion and hypertension and T2DM among South Asian adults [33]. Neighborhood social cohesion was defined as perceived connectedness and shared resources that allow individuals to act as a group; this was measured through a validated five-item scale [33]. The study found a negative association between social cohesion and prevalence of hypertension and T2DM; however, there was no statistical significance when adjusted for socioeconomic, psychosocial, and physiologic factors [33]. The highest level of neighborhood social cohesion was significantly associated with a lower prevalence of hypertension (OR 0.62, 95% CI 0.43–0.91) and type 2 diabetes (OR 0.57, 95% CI 0.35–0.93) [33]. However, when adjusted for social factors and health behaviors, the association of increased social cohesion with decreased prevalence of hypertension and diabetes remained but was not statistically significant [33]. Additionally, they examined if sex may moderate the association between social cohesion and health outcomes. For South Asian women, those with high levels of social cohesion had a lower prevalence of hypertension (OR 0.57, 95% CI 0.30–0.99) [33].

Lu et al. examined the relationship between perceived stress, social support, and hypertension among Chinese, Korean, and Vietnamese Americans [34]. Social support was roughly defined as a social network that provides positive experiences and stability in life especially when stressors arise [34]. Social support was measured on an 8-term scale quantifying the amount and type of social support, and perceived stress was measured on a 10-item version of the Perceived Stress Scale [34]. In the study, Chinese participants with high levels of perceived stress were more likely to have hypertension [34]. Chinese participants with high social support were less likely to have hypertension (OR 0.36, 95% CI 0.15–0.87) [34]. This trend was also observed in Koreans and Vietnamese but was not statistically significant [34].

### Health literacy

One study examined health literacy as a social factor. In Sentell et al., this was measured by a validated single-item of “How confident are you filling out medical forms by yourself?” The study by Sentell et al. examined health literacy and health status, which included diabetes, among Japanese, Filipino, Native Hawaiian, and other Asian American/Pacific Islander (AAPI) living in Hawaii [32]. Japanese (OR 1.78, 95% CI 1.00–3.16) and Filipino (OR 1.80, 95% CI 0.88–3.67) participants with low literacy had a higher prevalence of hypertension; this, however, was statistically significant for only Japanese participants [32].

## Discussion

Large observational studies suggest Asian-Americans may be disproportionately affected by CMD compared to whites, however little is known about disaggregated Asian groups in the U. S [35, 36]. Moreover, understanding the vital context of where Americans live, work and play can help us evaluate and develop interventions that target the social needs of varying groups. This review aimed to systematically assess the literature over the last 20 years to identify the major SDOH contributing to the burden of CMD among Asians in the U.S. Our review identified four major SDOH that may be affecting the CMD burden among Asians; these include acculturation, SES, social context, and health literacy. Acculturation, broadly defined as years living in the US, native country of birth, and English proficiency was the most common social factor across Asian subgroups. The majority of studies included South Asians, Chinese, and Filipinos..

Acculturation was the most commonly studied social factor examined for its influence on CMD. Generally, it appears that most groups see an association of CMD risk with years lived in the U.S. However, though data are limited, it appears that there is variation by groups with South Asians and Filipinos having increased CMD risk, but this is not observed in Yang’s study of Korean adults. One area that deserves further attention is the effect of English proficiency on CMD risk. It is well-studied that other groups with limited English proficiency may be at higher risk of poor health outcomes [37–40]. However, our study highlights a major gap in understanding how LEP may affect CMD health among Asian American subgroups. This is likely closely tied to health literacy, of which we only identified one study examining health literacy and CMD risk among Japanese, Filipino, and AAPI that did find an increased risk of hypertension among Japanese with low health literacy. Integrated measures of both English proficiency and health literacy that are Asian-subgroup specific, may help disentangle what the varying needs and gaps are in Asian populations.

Educational attainment was associated with a higher risk of hypertension among Koreans, Filipinos, and Chinese and a higher risk of diabetes among Chinese, South Asians, and Japanese [9, 26, 27, 31, 32]. This association varied in strength and significance. This may reflect varying patterns of immigration seen among these groups. For example, South Asians were more likely to immigrate to the U.S. after the 1965 immigrant act, which favored immigrants with professional degrees, thus 40% of Asian Indians for example had a Master’s degree or higher in 2015 [41]. Our findings on SES, which can often be linked to educational attainment warrants further attention. Unlike what is observed among American adults, SES was not associated with major differences in CMD risk for Chinese and South Asians. This may reflect the healthy immigrant effect, in which recent immigrants are found to be healthier despite lower socioeconomic status and health disparities [42]. More importantly, there are limited data on SES and its association with CMD for most Asian subgroups in the U.S. This represents a major area of future studies to put our findings into context for the diverse Asian subgroups in the U.S. Moreover, none of these studies delineated native-born vs. U.S.-born which could also be important in understanding SES and CMD risk. Lastly, there may be gender-specific findings that are lost in the aggregated data we have, that should be further studied.

We found limited observational data on social cohesion and social support among Asian subgroups. The MASALA study, the largest and only U.S.-based longitudinal cohort of South Asians collects data on social support and neighborhood contextual factors such as chronic psychological stress, perception of discrimination, and neighborhood environment [43]. Data from the first wave of the MASALA cohort did not show a link between social cohesion and CMDs, yet data collection and monitoring are ongoing. Future studies are needed to understand this relationship. Social support is a well-known influencer of CMD outcomes among various immigrant groups and other US-born populations [44–47]. Our study shows this may also be true for Chinese Americans as well. Several interventions study of Filipinos and Koreans though demonstrate that social support can aid in such areas as diabetes prevention, self-management, and physical activity [48, 49]. Yet, there are limited data examining social support and cohesion, among Asian subgroups and no standardized measures across groups, representing a major area for future investigation.

Our study has several limitations to consider. First, this study only included observational studies; while there are interventions that collect data on SDOH, our aim was to describe the current landscape of SDOH and CMD risk among Asian Americans, thus interventions were excluded. Second, some of the studies relied on self-reported data for both exposures and outcomes, which may limit the validity and reliability of the measures. This could have also led to some selection bias in the populations included in this review. For example, if the study outcomes of interest relied on self-report, yet potential study participants have limited access to care, they may not have been screened for CMDs, thus they would not be included and may bias the conclusions from this review. Of note, Seven of the 14 studies collected data, including blood assays and blood pressure, in a controlled lab setting. Third, many of the studies were conducted in regions of the U.S. with large Asian populations, which may limit the generalizability of the studies. Thus, the studies may not be representative of individuals not living in communities with large Asian American populations. Fourth, we excluded studies that included “Asian Americans” without providing granular detail on the study population, given the heterogeneity we hypothesized would exist by subgroups. Additionally, there may be limitations of our search strategy of using keywords, resulting in the potential exclusion of relevant studies that did not use the same keywords used in our search. Lastly, in order to narrow the scope of our study, we used a number of health outcomes that we broadly categorized as CMD but excluded intermediate outcomes such as coronary calcification, physical activity, and obesity, which may be precursors to CMD and are also likely influenced by the SDOHs. Further research may focus specifically on interventions that address SDOH among Asian subgroups and on other CMD-related outcomes.

## Conclusions

To our knowledge, this is the first systematic review examining the association of SDOH and CMD risk among specific Asian subgroups. While often considered the “model minority” our review demonstrates that important sociodemographic factors including acculturation, SES, and social context also disproportionally affect Asian subgroups [50]. However future studies that target specific Asian groups, especially those representing the majority of Asian groups in American (Asian Indians, Chinese, Filipino, Vietnamese, Korean, and Japanese) are needed. Additionally, better demographic data collection on Asian subgroups would promote identifying gaps and areas of improvement for specific subgroups. Future areas of study and major gaps in the current literature include health literacy, the effects of LEP among Asian subgroups, sex-specific variation, social support, and social cohesion and how they relate to CMD risk. Disentangling these social determinants of health for subpopulations of Asians can help develop tailored interventions and policies to support the health of Asian Americans in the U.S.

## Supplementary Information

**Additional file 1.** Keywords used in search strategy.

## Data Availability

Not applicable.

## Abbreviations

CMD: Cardiometabolic Disease
SDOH: Social Determinants of Health
BMI: Body Mass Index
SES: Socioeconomic Status
U.S.: United States
NHLBI: National Heart, Lung, and Blood Institute
CI: Confidence Interval
T2DM: Type II Diabetes

## References

[1] Profile: Asian American (2021). U.S. Department of Heath and Human Services Office of Minority Health.

[2] Race A (2021). The United States Census Bureau.

[3] Colby S, Ortman J (2015). Projections of the Size and Composition of the U.S: 2014-2060. The United States Census Bureau.

[4] Budiman A, Ruiz N (2021). Key facts about Asian Americans, a diverse and growing poepulation. Pew Research Center.

[5] Modern Immigration Wave Brings 59 Million to U.S (2015). Pew Research Center’s Hispanic Trends Project.

[6] Praveen PA, Kumar SR, Tandon N (2015). Type 2 diabetes in youth in South Asia. Curre Diabetes Rep.

[7] Vikram NK, Pandey RM, Misra A, Sharma R, Rama Devi J, Khanna N (2003). Non-obese (body mass index < 25 kg/m2) Asian Indians with normal waist circumference have high cardiovascular risk. Nutrition.

[8] Raji A, Seely EW, Arky RA, Simonson DC (2001). Body fat distribution and insulin resistance in healthy Asian Indians and Caucasians. J Clin Endocrinol Metab.

[9] Shah AD, Vittinghoff E, Kandula NR, Srivastava S, Kanaya AM (2015). Correlates of prediabetes and type II diabetes in US south Asians: findings from the mediators of atherosclerosis in south Asians living in America (MASALA) study. Ann Epidemiol.

[10] Flowers E, Lin F, Kandula NR, Allison M, Carr JJ, Ding J (2019). Body composition and diabetes risk in south Asians: findings from the MASALA and MESA studies. Diabetes Care.

[11] Santos VA, Palaniappan LS, Aggarwal NT, Gupta M, Khandelwal A, Krishnan AV (2018). Atherosclerotic Cardiovascular Disease in South Asians in the United States: Epidemiology, Risk Factors, and Treatments: A Scientific Statement From the American Heart Association. Circulation.

[12] Marmot M, Friel S, Bell R, Houweling TAJ, Taylor S (2008). Closing the gap in a generation: health equity through action on the social determinants of health. Lancet.

[13] Havranek EP, Mujahid MS, Barr DA, Blair IV, Cohen MS, Cruz-Flores S (2015). Social determinants of risk and outcomes for cardiovascular disease | circulation. Circulation.

[14] Palaniappan LP, Araneta MRG, Assimes TL, Barrett-Connor EL, Carnethon MR, Criqui MH (2010). Call to action: cardiovascular disease in Asian Americans a science advisory from the American Heart Association. Circulation.

[15] Page MJ, McKenzie JE, Bossuyt PM, Boutron I, Hoffmann TC, Mulrow CD (2021). The PRISMA 2020 statement: an updated guideline for reporting systematic reviews. BMJ.

[16] Veritas Health Innovation. Covidence systematic review software.

[17] Morgan RL, Whaley P, Thayer KA, Schunemann HJ (2018). Identifying the PECO: a framework for formulating good questions to explore the association of environmental and other exposures with health outcomes. Environ Int.

[18] Healthy People (2030). Social determinants of health. U.S. Department of Health and Human Services.

[19] Solar O, Irwin A (2010). A conceptual framework for action on the social determinants of health.

[20] Santana S, Brach C, Harris L, Ochiai E, Blakey C, Bevington F, Kleinman D, Pronk N. Updating Health Literacy for Healthy People 2030: Defining Its Importance for a New Decade in Public Health. J Public Health Manag Pract. 2021;27(Suppl 6):S258–S264. 10.1097/PHH.0000000000001324. 10.1097/PHH.0000000000001324 PMC8435055 33729194

[21] National Heart Lung and Blood Institute (NHLBI). Study Quality Assessment Tools. https://www.nhlbi.nih.gov/health-topics/study-quality-assessment-tools.

[22] Lee JR, Maruthur NM, Yeh HC (2020). Nativity and prevalence of cardiometabolic diseases among U.S. Asian immigrants. J Diabetes Complications.

[23] Bayog MLG, Waters CM (2018). Nativity, chronic health conditions, and health behaviors in Filipino Americans. J Transcult Nurs.

[24] Ursua RA, Islam NS, Aguilar DE, Wyatt LC, Tandon SD, Abesamis-Mendoza N (2013). Predictors of hypertension among filipino immigrants in the northeast US. J Community Health.

[25] Ma GX, Lee M, Bhimla A, Tan Y, Gadegbeku CA, Yeh MC (2017). Risk assessment and prevention of hypertension in Filipino Americans. J Community Health.

[26] Yi SS, Thorpe LE, Zanowiak JM, Trinh-Shevrin C, Islam NS (2016). Clinical characteristics and lifestyle behaviors in a population-based sample of Chinese and south Asian immigrants with hypertension. Am J Hypertens.

[27] Kim MT, Kim KB, Juon H (2000). Prevalence and factors associated with high blood pressure in Korean Americans. Ethnicity Disease.

[28] Huang ZJ, Zheng C (2015). Type 2 diabetes among 6 asian ethnic groups in California: the nexus of ethnicity, gender, and generational status. J Health Care Poor Underserved.

[29] Kandula NR, Diez-Roux AV, Chan C, Daviglus ML, Jackson SA, Ni H (2008). Association of acculturation levels and prevalence of diabetes in the multi-ethnic study of atherosclerosis (MESA). Diabetes Care.

[30] Yang EJ, Chung HK, Kim WY, Bianchi L, Song WO (2007). Chronic diseases and dietary changes in relation to Korean Americans’ length of residence in the United States. J Am Diet Assoc.

[31] Boykin S, Diez-Roux AV, Carnethon M, Shrager S, Ni H, Whitt-Glover M (2011). Racial/ethnic heterogeneity in the socioeconomic patterning of CVD risk factors: in the United States: the multi-ethnic study of atherosclerosis. J Health Care Poor Underserved.

[32] Sentell T, Baker KK, Onaka A, Braun K (2011). Low health literacy and poor health status in Asian americans and pacific islanders in Hawai’i. J Health Commun.

[33] Lagisetty PA, Wen M, Choi H, Heisler M, Kanaya AM, Kandula NR (2016). Neighborhood social cohesion and prevalence of hypertension and diabetes in a south Asian population. J Immigr Minor Health.

[34] Lu X, Juon HS, He X, Dallal CM, Wang MQ, Lee S (2019). The association between perceived stress and hypertension among Asian Americans: does social support and social network make a difference?. J Community Health.

[35] Cheng YJ, Kanaya AM, Araneta MRG, Saydah SH, Kahn HS, Gregg EW (2019). Prevalence of diabetes by Race and ethnicity in the United States, 2011-2016. JAMA.

[36] Palaniappan L, Wang Y, Fortmann SP (2004). Coronary heart disease mortality for six ethnic groups in California, 1990-2000. Ann Epidemiol.

[37] Sentell T, Braun KL (2012). Low health literacy, limited English proficiency, and health status in Asians, Latinos, and other racial/ethnic groups in California. J Health Commun.

[38] Kim EJ, Kim T, Paasche-Orlow MK, Rose AJ, Hanchate AD (2017). Disparities in hypertension associated with limited English proficiency. J Gen Intern Med.

[39] Fiscella K, Franks P, Doescher MP, Saver BG (2002). Disparities in health care by race, ethnicity, and language among the insured: findings from a national sample. Med Care.

[40] DuBard CA, Gizlice Z (2008). Language spoken and differences in health status, access to care, and receipt of preventive services among US Hispanics. Am J Public Health.

[41] Educational attainment of Indian population in the U.S.,2015. 2017. https://www.pewresearch.org/social-trends/chart/educational-attainment-of-indian-population-in-the-u-s/. Accessed 8 Jun 2021.

[42] Markides KS, Rote S (2019). The healthy immigrant effect and aging in the United States and other Western countries. Gerontologist.

[43] Kanaya AM, Herrington D, Vittinghoff E, Ewing SK, Liu K, Blaha MJ (2014). Understanding the high prevalence of diabetes in U.S. south Asians compared with four racial/ethnic groups: the MASALA and MESA studies. Diabetes Care.

[44] Lee AA, Piette JD, Heisler M, Rosland AM. Diabetes Distress and Glycemic Control: The Buffering Effect of Autonomy Support From Important Family Members and Friends. Diab Care. 2018;41(6):1157–1163. 10.2337/dc17-2396. 10.2337/dc17-2396 PMC5961390 29599295

[45] Lee AA, Piette JD, Heisler M, Rosland AM (2018). Diabetes distress and glycemic control: the buffering effect of autonomy support from important family members and friends. Diabetes Care.

[46] Freeborne N, Simmens SJ, Manson JAE, Howard BV, Cené CW, Allison MA (2019). Perceived social support and the risk of cardiovascular disease and all-cause mortality in the Women’s health initiative observational study. Menopause.

[47] Lett HS, Blumenthal JA, Babyak MA, Strauman TJ, Robins C, Sherwood A (2005). Social support and coronary heart disease: epidemiologic evidence and implications for treatment. Psychosom Med.

[48] Kim MT, Kim KB, Huh B, Nguyen T, Han H-R, Bone LR (2015). The effect of a community-based self-help intervention: Korean Americans with type 2 diabetes. Am J Prev Med.

[49] Leake AR, Bermudo VC, Jacob J, Jacob MR, Inouye J (2012). Health is wealth: methods to improve attendance in a lifestyle intervention for a largely immigrant Filipino-American sample. J Immigr Minor Health.

[50] Yi SS, Kwon SC, Sacks R, Trinh-Shevrin C (2016). Commentary: persistence and health-related consequences of the model minority stereotype for Asian Americans. Ethn Dis.

