# Supplementary material for: The social determinants of health associated with cardiometabolic diseases among Asian American subgroups: a systematic review
Source: BMC Health Serv Res. 2022 Feb 25;22:257. doi: 10.1186/s12913-022-07646-7 (PMC8876533; doi:10.1186/s12913-022-07646-7)
Supplement: Supplementary file 1 — Additional file 1. Keywords used in search strategy. [file 12913_2022_7646_MOESM1_ESM.docx]

**Additional File 1**. Keywords used in search strategy

| **Database** | **Search Query** |
| --- | --- |
| PubMed | ("Asian Americans"[Mesh]) AND ("Diabetes Mellitus"[Mesh] OR "Hypertension"[Mesh] OR "Coronary Artery Disease"[Mesh] OR "Stroke"[Mesh]) |
|  | ("Emigrants and Immigrants"[Mesh]) AND ("Asian Continental Ancestry Group"[Mesh]) AND ("Diabetes Mellitus"[Mesh] OR "Hypertension"[Mesh] OR "Coronary Artery Disease"[Mesh] OR "Stroke"[Mesh]) |
| Web of Science | TS = (asian american) AND TS = (cardiometabolic disease OR diabetes OR hypertension OR coronary artery disease OR stroke) AND TS = (determinant) |
| Embase | ('asian american'/exp OR 'asian american' OR 'asian americans' OR 'chinese american' OR 'japanese american' OR 'american, asian') AND ('social determinants of health'/exp OR 'social determinant' OR 'social determinants' OR 'social determinants of health' OR 'social determining factor' OR 'social factors determining health' OR 'social health determinant') AND ('diabetes mellitus'/exp OR 'diabetes' OR 'diabetes mellitus' OR 'diabetic' OR 'hypertension'/exp OR 'htn (hypertension)' OR 'acute hypertension' OR 'arterial hypertension' OR 'blood pressure, high' OR 'cardiovascular hypertension' OR 'controlled hypertension' OR 'endocrine hypertension' OR 'high blood pressure' OR 'high renin hypertension' OR 'hypertension' OR 'hypertensive disease' OR 'hypertensive effect' OR 'hypertensive response' OR 'neurogenic hypertension' OR 'preexistent hypertension' OR 'refractory hypertension' OR 'salt high blood pressure' OR 'salt hypertension' OR 'secondary hypertension' OR 'systemic hypertension' OR 'coronary artery disease'/exp OR 'coronary artery disease' OR 'coronary disease' OR 'multivessel coronary artery disease' OR 'cerebrovascular accident'/exp OR 'cva' OR 'accident, cerebrovascular' OR 'acute cerebrovascular lesion' OR 'acute focal cerebral vasculopathy' OR 'acute stroke' OR 'apoplectic stroke' OR 'apoplexia' OR 'apoplexy' OR 'blood flow disturbance, brain' OR 'brain accident' OR 'brain attack' OR 'brain blood flow disturbance' OR 'brain insult' OR 'brain insultus' OR 'brain ischaemic attack' OR 'brain ischemic attack' OR 'brain vascular accident' OR 'cerebral apoplexia' OR 'cerebral insult' OR 'cerebral stroke' OR 'cerebral vascular accident' OR 'cerebral vascular insufficiency' OR 'cerebro vascular accident' OR 'cerebrovascular accident' OR 'cerebrovascular arrest' OR 'cerebrovascular failure' OR 'cerebrovascular injury' OR 'cerebrovascular insufficiency' OR 'cerebrovascular insult' OR 'cerebrum vascular accident' OR 'cryptogenic stroke' OR 'ischaemic cerebral attack' OR 'ischaemic seizure' OR 'ischemic cerebral attack' OR 'ischemic seizure' OR 'stroke') |
